# Ethanol Regulates Presynaptic Activity and Sedation through Presynaptic Unc13 Proteins in *Drosophila*

**DOI:** 10.1523/ENEURO.0125-18.2018

**Published:** 2018-06-08

**Authors:** Shiyu Xu, Satyabrata Pany, Kevin Benny, Khadeeja Tarique, Ola al-Hatem, Kathleen Gajewski, J. Leigh Leasure, Joydip Das, Gregg Roman

**Affiliations:** 1 Department of Biology and Biochemistry, University of Houston, Houston, TX 77204; 2 Biology of Behavior Institute, University of Houston, Houston, TX 77204; 3 Department of Pharmacological and Pharmaceutical Sciences, University of Houston, Houston, TX 77204; 4 Department of Psychology, University of Houston, Houston, TX 77204; 5 Department of Biology, University of Mississippi, MS 38677

**Keywords:** *Drosophila*, ethanol, Munc13-1, presynaptic, resistance, tolerance

## Abstract

Ethanol has robust effects on presynaptic activity in many neurons, however, it is not yet clear how this drug acts within this compartment to change neural activity, nor the significance of this change on behavior and physiology *in vivo*. One possible presynaptic effector for ethanol is the Munc13-1 protein. Herein, we show that ethanol binding to the rat Munc13-1 C1 domain, at concentrations consistent with binge exposure, reduces diacylglycerol (DAG) binding. The inhibition of DAG binding is predicted to reduce the activity of Munc13-1 and presynaptic release. In *Drosophila*, we show that sedating concentrations of ethanol significantly reduce synaptic vesicle release in olfactory sensory neurons (OSNs), while having no significant impact on membrane depolarization and Ca^2+^ influx into the presynaptic compartment. These data indicate that ethanol targets the active zone in reducing synaptic vesicle exocytosis. *Drosophila*, haploinsufficent for the Munc13-1 ortholog *Dunc13*, are more resistant to the effect of ethanol on presynaptic inhibition. Genetically reducing the activity of *Dunc13* through mutation or expression of RNAi transgenes also leads to a significant resistance to the sedative effects of ethanol. The neuronal expression of *Munc13-1* in heterozygotes for a *Dunc13* loss-of-function mutation can largely rescue the ethanol sedation resistance phenotype, indicating a conservation of function between *Munc13-1* and *Dunc13* in ethanol sedation. Hence, reducing *Dunc13* activity leads to naïve physiological and behavioral resistance to sedating concentrations of ethanol. We propose that reducing *Dunc13* activity, genetically or pharmacologically by ethanol binding to the C1 domain of Munc13-1/Dunc13, promotes a homeostatic response that leads to ethanol tolerance.

## Significance Statement

At relatively low concentrations, ethanol inhibits the activity of many presynaptic termini (Liu and Hunt, 1999). Homeostatic changes in presynaptic activity are proposed to underlie the formation of functional tolerance (Koob and Bloom, 1988; Ghezzi and Atkinson, 2011). We do not currently know where ethanol binds to bring about changes in presynaptic activity and homeostasis. We now show that ethanol will bind to the C1 domain of the Munc13-1 protein at intoxicating concentrations and inhibit the binding of diacylglycerol (DAG). Reducing the activity of the *Drosophila* Dunc13 ortholog leads to a homeostatic change that promotes behavioral and physiologic resistance to intoxicating levels of ethanol. Hence, Unc13 proteins are likely sites for ethanol’s action within the presynaptic compartment that bring about tolerance.

## Introduction

Addiction to alcohol remains one of the most significant mental health problems throughout of the world. A major challenge is to understand how ethanol changes behavior and the brain during the descent into addiction. A promising approach is to examine endophenotypes for this disease in model systems (Dick et al., 2006; Salvatore et al., 2015). One major endophenotype for the development of alcoholism is the naïve behavioral sensitivity to ethanol, wherein the sons of alcoholics there is a fourfold increase in the likelihood of alcoholism among those with a reduced naïve sensitivity to the intoxicating effects of alcohol (Schuckit, 1980, 1994; Schuckit et al., 1996). The naïve resistance to ethanol intoxication may be mechanistically related to the formation of tolerance that follows exposure to high concentrations of ethanol (Harris et al., 2008; Mayfield et al., 2008).

During binge alcohol exposure, ethanol creates widespread and long-lasting changes in neural activity, altering both presynaptic and postsynaptic activity. An effect of ethanol on presynaptic release is seen at concentrations below 100 mM, where this drug typically produces its sedative effects (Diamond and Gordon, 1997). A single amino acid polymorphism in the Munc-18 active zone protein and its homolog unc-18 were found to generate strong resistance to ethanol sedation in mouse and *Caenorhabditis elegans*, respectively (Fehr et al., 2005; Graham et al., 2009). This mutation in Unc18 also decreased the frequency of synaptic vesicle release (Graham et al., 2009). The loss of synaptic vesicle proteins Rab-3A and its homolog Rab-3 also results in the resistance to ethanol sedation in both *C. elegans* and mouse (Kapfhamer et al., 2008). Ethanol inhibits presynaptic vesicle release in rat hippocampal slices induced by extracellular K^+^ triggered depolarization and dependent on voltage-gated calcium channel activity (Maldve et al., 2004). Since Munc-18, Rab-3A, and voltage-gated channels are essential for presynaptic exocytosis, ethanol may alter presynaptic activity by changing the probability of synaptic vesicle fusion. The mechanism by which ethanol effects reduction in presynaptic activity is unknown, but one candidate is the *slowpoke* large conductance BK channel. The activity of the *slowpoke* BK channel has an important role in ethanol sedation and tolerance in both *Drosophila* and *C. elegans* (Davies et al., 2003; Cowmeadow et al., 2005, 2006). Ethanol can directly bind to BK channels and the binding is essential for ethanol sedation in *C. elegans* (Bukiya et al., 2014; Davis et al., 2014). However, BK channel is expressed both presynaptically and postsynaptically, and it is not yet clear how much of the change in presynaptic activity is attributed to ethanol binding to presynaptic BK channels (Sailer et al., 2006).

Munc13-1 is also an alcohol binding protein and a strong candidate for producing an effect of alcohol in the presynaptic compartment (Das et al., 2013). Munc13-1 is an active-zone protein essential for synaptic vesicle fusion (Betz et al., 1997, 1998; Rhee et al., 2002). Munc13-1 directly interacts with and coordinates several other members of vesicle fusion machinery at presynaptic action zones in the mammalian brain, including syntaxin, RIM, Munc18, and voltage-gated calcium channels (VGCCs), (Rizo and Xu, 2015). The C1 domain of Munc13-1 binds diacylglycerol (DAG), which promotes membrane localization of this protein and lowers the energy barrier for vesicle fusion, facilitating synaptic vesicle release (Basu et al., 2007). Ethanol binds to the Munc13-1 C1 domain *in vitro* at concentrations below 100 mM (Das et al., 2013). Reduction in the level of *Dunc13*, the *Drosophila* ortholog of *Munc13-1*, results in flies that self-administer ethanol at significantly higher levels than wild-type controls (Das et al., 2013). Herein, we demonstrate that ethanol inhibits DAG binding to the Munc13-1 C1 domain at concentration as low as 25 mM. Furthermore, we show in *Drosophila* that ethanol impairs synaptic vesicle release in excitatory neurons downstream of Ca^2+^ influx into the active zone and that the reduction in *Dunc13* produces a behavioral and physiologic resistance to sedative effects of ethanol.

## Materials and Methods

### Fly strains

All flies were raised on standard cornmeal food at 25°C on a 12/12 h light/dark cycle. All the stocks were out-crossed into the Canton-S background for a minimum of six generations before behavioral analysis. *Dunc13^P84200^*/*ci^D^*(FBst0300878) was generously provided by the Kyoto Stock Center. *Dunc13^KK101383^* RNAi line (RRID: FlyBase_FBst0479208) was provided by Vienna Drosophila Resource Center. The *elav*-Gal4 (FBst0008760, RRID: BDSC_8760), UAS-*Arclight* (RRID: BDSC 51056, FBst0051056), UAS-*GCaMP5* (RRID: FlyBase_FBst0042037) and *Dunc13^JF02440^* RNAi line (RRID: BDSC_29548) were provided by Bloomington Drosophila Stock Center. The *n-syb*-Gal4 was generously provided by Herman Dierick (Baylor College of Medicine). The *Or42b*-Gal4 was kindly provided by Scott Pletcher (University of Michigan). The *tublin*-Gal80^ts2^, *ry^506^*, and UAS-*pHluorin* were generously provided by Ronald L. Davis (Scripps, FL).

The Munc13.1-EGFP cDNA was digested from the pBRETU vector (Roman et al., 1999; Das et al., 2013), and cloned into pUAST-attB vector (Bischof et al., 2007). The Munc13.1-pUAST-attB vector was recombined into attP40 in second chromosome, and the position and orientation were confirmed by PCR.

For the ethanol sedation assay, the *Dunc13^P84200^/+* genotype was generated by crossing virgin females of *ry^506^*, with *w* ^+^; +; *ry^506^*; *Dunc13^P84200^*/*ci^D^*males. For the functional complementation of *Dunc13^P84200^*, the experimental genotype was generated by crossing virgin females of *w* ^+^; *tublin*-Gal80^ts^; *ry^506^*; *Dunc13*/*ci^D^*, with *w* ^+^; UAS-*Munc13-1* (or UAS-*dunc13A*); *elav*-Gal4;+ males. For optical imaging, the genotype Or42b-Gal4/+; UAS- pHluorin (or *Arclight*, *GCaMP5*)/+ was generated by crossing virgin females of Or42b-Gal4, with UAS-*pHluorin* (or *Arclight*, *GCaMP5*) males, and the genotype Or42b-Gal4/+; UAS- pHluorin/+; *Dunc13^P84200^*/+ was generated by crossing virgin females of Or42b-Gal4;; *Dunc13^P84200^*/*ci^D^*, with UAS-*pHluorin* males.

### Ethanol sedation

The ethanol loss of righting reflex (LOR) assay was performed as previously described (van der Linde et al., 2014). Female flies were collected and placed in vials containing fresh food (1 *n* = 30 flies per vial) for 24 h before behavioral testing. They were then transferred to the empty plastic vials of the test apparatus and exposed to a stream of ethanol vapor (50% unless noted) at a flow of 250 ml/min for 1 h to test for ethanol sensitivity. The ethanol vapor was produced by bubbling fresh air through 100% ethanol and pure water and mixing the two kinds of vapors. The % ethanol refers to the percentage of the final air/ethanol stream made up of the air bubbled through ethanol. The percentage of flies sedated in each vial was recorded at 5-min intervals. A fly was counted as being sedated if it had fallen onto its back or side and could not right itself. The time to 50% LOR was calculated for each exposure tube by linear interpolation of the two time points around the median and then averaged over the number of tubes (Ojelade et al., 2015).

### Ethanol metabolism and absorption

The ethanol metabolism and absorption assay has been described before (Urizar et al., 2007). To study ethanol absorption, 30 female flies were exposed to 50% ethanol vapor at a 250 ml/min flow rate for 0, 20, 40, or 60 min. Immediately after exposure, flies were frozen in liquid nitrogen and homogenized in 200 μl of 50 mM Tris-HCl, pH 7.5. The homogenate was then centrifuged at 15,000 × *g* at 4°C for 20 min, and the supernatant was collected. The ethanol concentration in the supernatant was measured using the Ethanol Assay kit (catalog #MAK076, Sigma-Aldrich). To determine whether equivalent amounts of flies were assayed for each genotype, the protein concentration in the fly extracts was measured using the DC protein assay (Bio-Rad Laboratories). Ethanol metabolism was examined by exposing flies to 50% ethanol vapor for 60 min and allowing flies to recover for a period of 0, 30, 60, or 120 min. The ethanol concentration in fly extracts was measured at the end of each recovery period.

### Optical imaging and ethanol injection

Optical imaging was performed on live flies as previously described with modifications (Zhang and Roman, 2013). Each female fly was fixed on a pipette tip and its head was secured to the tip opening with wax. The cuticle on the top of head was removed and covered with a piece of plastic wrap.

Before injection, each dissected female fly was checked through gently touching the tip of its foreleg with forceps. If the fly’s femur did not show any movement, it would be discarded since it might be dead or close to dying. To inject ethanol, a small piece of pipette tip which contained fixed fly was removed to expose its scutellum. An insect pin (catalog #26002-10, Fine Science Tools) was inserted between scutum and scutellum to make a hole. A glass pipette (top diameter ∼20 μm), was inserted into the hole. Then an injector (MINJ-PD, Tritech Research) linked with the glass pipette was turned on to inject ∼0.1 μl ethanol solution into fly. The injected solution was Ringer’s solution plus 15% ethanol.

After 10 min, the injected fly was imaged under an upright confocal microscope with a 20× objective (SP8, Leica). Ethyl acetate was diluted in mineral oil to 1% and delivered with an air flow at a rate of 400 ml/min bubbled through the mineral oil. The delivery of odorants was accomplished with a CS55 stimulus controller (Syntech), which added the odor to an airstream. Flies were exposed to 1-min air and then 3-s odor; there were no mechanical disturbances during the transition from air to odor.

After the imaging, each fly was taken out of the pipette tip to check for viability. Most of the flies exhibited leg movements. However, if a fly’s legs showed no movement, it was considered dead, and the data were discarded.

### Biochemistry

The Munc13-1 C1 domain in pGEX-KG vector was kindly provided by Dr. Josep Rizo (University of Texas Southwestern Medical Center at Dallas, TX). Site-specific mutations in the Munc 13-1 C1 domain were introduced with PCR by presenting the mutation in oligonucleotide primers using the he QuikChange II Site-Directed Mutagenesis kit (Agilent Technologies). The Munc 13-1 C1 domain wild-type or the mutant fused with glutathione *S*-transferase (GST) were expressed in BL21 DE3 gold *Escherichia coli* (Agilent Technologies). Munc13.1C1 and its E582A mutant were purified following the methods described earlier (Das et al., 2013). Fluorescence resonance energy transfer (FRET) between Munc13.1 C1 and dansyl-DAG were determined using a PTI fluorimeter (Photon Technology Instruments; Pany and Das, 2015). Samples containing Munc13.1C1 (1 μM) and dansyl-DAG (1 μM) in buffer (50 mM Tris, 100 mM NaCl, and 5 mM ZnSO_4_; pH 8) were incubated for 30 min at 25°C. For measuring the effect of ethanol, mixtures were incubated with ethanol (25 and 50 mM) for 30 min at 25°C. Samples were then excited at 280 nm, and emission spectra were recorded from 300 to 550 nm. Emission maxima of Munc13.1 C1 and dansyl-DAG were found to be at 337 and 500 nm, respectively. Relative FRET indices were calculated from fluorescence intensity at 500 nm using formula: [(Fi + Munc13.1C1) − (Fi, −Munc13.1C1)] − [(F0 + Munc13.1C1) − (F0− Munc13.1C1)], where Fi + Munc13.1C1 and Fi − Munc13.1C1 are the intensities of Dansyl-DAG in the presence and absence of Munc13.1C1, respectively, and F0+ Munc13.1C1 and F0− Munc13.1C1 are fluorescence intensities of the buffer solution in the presence or absence of Munc13.1C1, respectively. The change in fluorescence intensities for each concentration of alcohol was normalized using the equation: (1-F/F0) × 100, where F and F0 are intensities of dansyl-DAG plus Munc13.1C1 in the presence or in the absence of alcohol.

### Experimental design and statistical analysis

The differences between two groups were tested with two-tailed, unpaired Student’s *t* tests; the differences among multi-group data were tested with two-way ANOVA (treatment × genotype), followed by Bonferroni-Dunn *post hoc* tests. Planned within genotype comparisons within these data were analyzed by Student’s *t* tests. For each fly in the optical imaging experiments, the maximum fluorescent intensity change ratio before and after stimulation were calculated first. Then the ratio of vehicle-injected flies and ethanol-injected flies was analyzed by two-tailed, unpaired Student’s *t* tests. For *t* test and ANOVA, α = 0.05.

## Results

### Ethanol binding to the C1 domain inhibits DAG binding

**Figure 1.**
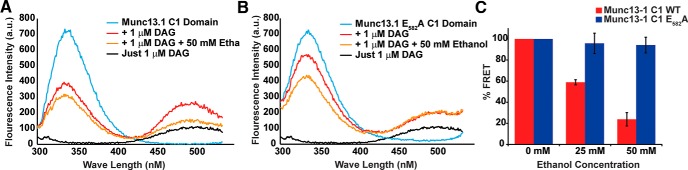
Ethanol binding to the C1 domain inhibits DAG binding. ***A***, Spectral emission after excitation at 290 nm reveals FRET between the Munc13-1 C1 domain and dansyl-DAG (red trace). The C1 domain emission peaks at ∼335 nm (cyan trace), while the dansyl-DAG emission peak is found at 500 nm (black trace). This FRET is disrupted by 50 mM ethanol (green trace). ***B***, Spectral emission of the Munc13-1 E_582_A mutation after excitation by 290-nm light is shown. ***C***, The binding of dansyl-DAG as revealed by FRET emission at 500 nm is reduced at both 25 and 50 mM ethanol concentrations. The E_582_A C1 domain mutation fails to bind ethanol (Das et al., 2013). Ethanol does not inhibit dansyl-DAG binding to the E_582_A Munc13-1 C1 domain (*n* = 3 each; *p < 0.05, ***p < 0.001). Error bars are standard error of the mean (SEM).

Ethanol may affect sedation by binding to Munc13-1/Dunc13 and directly changing the activity of these regulators of vesicle priming. Previously, ethanol was found to bind the E_582_ residue in the C1 DAG binding domain of Munc13-1 (Das et al., 2013). Since the distance between the E_582_ ethanol binding residue and the DAG binding site, His_567_, is only 8.8 A, ethanol binding to the C1 domain may impact DAG binding (Das et al., 2013). To investigate this question, we measured the effect of ethanol on the ability of the Munc13-1 C1 domain to bind DAG *in vivo*. FRET between the C1 domain and dansyl-DAG was used to quantify the relative amounts of DAG bound to the C1 domain *in vitro*. C1 domain fluorescence at ∼340 nm excited the dansyl-DAG and the dansyl-DAG emission was quantified at 500 nm (Fig. 1*A*,*B*). Overall, changing ethanol concentration and genotype of the C1 domain had a significant effect dansyl-DAG binding (*F*_(3,14)_ = 10.321, *p* < 0.001; genotype, *t* = 4.058, *p* = 0.001). Ethanol concentrations of 25 and 50 mM significantly reduced FRET between the wild-type Munc13-1 C1 domain and dansyl-DAG (*t* = 16.97 and *t* = 12.121, respectively, *p* < 0.001 for both). However, FRET between the Munc13-1E_582_A and dansyl DAG was not significantly altered by 25 and 50 mM ethanol (*t* = 0.439, *p* = 0.684 and *t* = 0.781, *p* = 0.479, respectively). This resistance to ethanol meant that the mutant E*_582_*A C1 domain was significantly less sensitive to the effects of ethanol at both 25 and 50 mM concentrations as compared to the wild-type C1 domain (*t* = 3.68, *p* = 0.021 and *t* = 7.21, *p* = 0.002; Fig. 1*C*). Hence, ethanol binding to the C1 domain reduces the affinity for DAG, presumably leading to reduced Munc13-1 activity. This inhibition relies on ethanol’s interaction with the C1 domain at E_582_.

### Ethanol inhibits presynaptic vesicle release in *Drosophila*

If the ethanol-induced inhibition of Munc13-1 activity is an important mechanism for ethanol effects on presynaptic activity, then ethanol should impact synaptic vesicle release *in vivo*, without impacting preceding presynaptic activation events. To examine this prediction, we measured the effect of ethanol on presynaptic activity in *Drosophila* of the OR42b olfactory sensory neurons (OSNs) using optical physiology techniques. The OR42b neurons are excitatory cholinergic neurons that are strongly activated by ethyl acetate (Hallem and Carlson, 2006). The OR42b OSN axons converge in the DM1 glomeruli (Gao et al., 2000; Couto et al., 2005). The *Or42b*-Gal4 line was used to drive three different genetically encoded sensors: ArcLight to measure changes in membrane depolarization (Cao et al., 2013); G-CaMP5 to measure Ca^2+^ influx into the presynaptic compartment (Akerboom et al., 2012); and synapto-pHluorin to measure synaptic vesicle fusion (Yuste et al., 2000; Reiff et al., 2005). These changes in fluorescence were measured in the DM1 glomeruli of the antennal lobe after a 3-s presentation of ethyl acetate. To measure the effect of ethanol on the physiologic changes in the OR42b OSN, we injected either 0.1 μl vehicle or 15% ethanol before imaging each fly. The ethanol-injected flies stopped normal leg movements, and appeared completely sedated for a period of ∼20 min. The flies were all imaged during this period of sedation. Vehicle-injected flies continued normal leg movements throughout the imaging period. Flies that did not recover leg movements after 20 min were excluded from the analysis.

**Figure 2.**
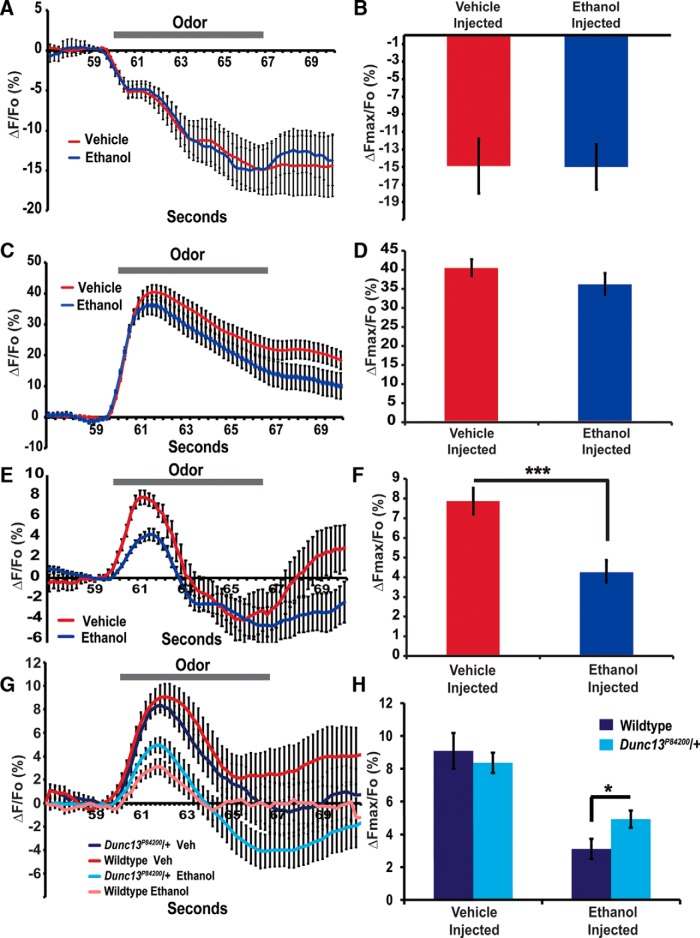
Ethanol specifically inhibits presynaptic vesicle release at excitatory synapses. ***A***, ***B***, Presynaptic activity was elicited by ethyl acetate delivered to the antennae and imaged within the DM3 glomeruli. Flies were imaged with ArcLight to measure membrane depolarization, which leads to a decrease in the maximal fluorescence (ΔFmax). Intoxication due to ethanol injection did not impact measured depolarization as measured by % ΔFmax/Fo (*p* > 0.05, *n* = 17). ***C***, ***D***, Flies were also imaged with G-CaMP5 to indicate the intracellular Ca^2+^ concentration. Increasing Ca^2+^ leads to an increase in fluorescence. Intoxication due to ethanol injection did not impact measured Ca^2+^ influx as measured by % ΔFmax/Fo (*p* > 0.05, *n* = 15). ***E***, ***F***, Flies were imaged with pHluorin to exhibit the presynaptic vesicle release. Intoxication due to ethanol injection significantly reduced synaptic vesicle fusion as measured by % ΔFmax/Fo (***p < 0.001, *n* = 17). ***G***, ***H***, In vehicle-injected flies, *Dunc13^P84200^*/+ heterozygotes do not show a significant reduction in synaptic vesicle release (*p* > 0.05, *n* = 19); however, they showed less ethanol-induced depression of synaptic release (*p < 0.05, *n* = 25), after 15% ethanol was injected. All error bars are SEMs.

Ethanol sedated flies did not have any detectable changes in ethyl acetate-induced membrane depolarizations within the presynaptic compartments of OR42b OSNs compared to vehicle-injected flies (*t* = 0.198, *p* = 0.845; Fig 2*A*,*B*). Similarly, we failed to detect a significant change in ethyl acetate induced Ca^2+^ influx into the OR42b OSN presynaptic compartment after ethanol sedation (*t* = 0.744, *p* = 0.464; Fig. 2*C*,*D*). In contrast, a significant decrease in the ethyl acetate elicited synaptic vesicle fusion was detected in the ethanol sedated flies (*t* = 4.129, *p* = 0.0002; Fig 2*E*,*F*). Together, these data that indicate that intoxicating levels of ethanol inhibits synaptic vesicle fusion largely independent of the incoming action potentials and resulting Ca^2+^ influx into the presynaptic compartment. These data are consistent with a role for ethanol-induced inhibition of Dunc13 activity, leading to a reduction in presynaptic release. It remains possible that ethanol may still impact Ca^+2^ influx within the presynaptic compartment in other time scales, such as minutes, but that we were unable to detect this effect. Munc13-1 enhances the function of presynaptic VGCC through direct interactions between these channels and the Munc13 C2B domain (Calloway et al., 2015). Hence, it remains possible that ethanol may subtly inhibit VGCC and presynaptic activity by inhibiting the binding between Munc13-1 and DAG.

Lastly, we asked if the inhibition of presynaptic vesicle fusion in response to ethanol was altered in the behaviorally resistant *Dunc13^P84200^*/*+* flies. The *Dunc13^P84200^* allele is a loss-of-function mutant with a P-element insertion into the *Dunc13* locus (Aravamudan et al., 1999). Homozygotes for this allele fail to display neurotransmission in the embryonic neuromuscular junction and die late in embryogenesis (Aravamudan et al., 1999). The heterozygotes have ∼50% wild-type levels of *Dunc13* mRNA (Das et al., 2013). In this experiment, there was a significant difference between groups (*F*_(2,81)_ = 21.474, *p* < 0.0001). As previously shown, injection of ethanol decreased the vesicle fusion in the OR42b OSNs elicited by ethyl acetate, however, the *Dunc13^P84200^*/*+* flies were less sensitive to this presynaptic inhibition as compared to wild-type controls (*t* = 2.224, *p* = 0.031; Fig 2*G*,*H*). Naïve *Dunc13^P84200^*/*+* heterozygotes are physiologically more resistant to ethanol’s inhibition of presynaptic activity.

### Dunc13 activity modulates ethanol sedation

To examine whether the Dunc13-ethanol interaction impacts behavioral responses to this drug, we further examined the effect of reducing the activity of the *Dunc13* on behavioral sensitivity to ethanol. *Dunc13^P84200^*/*+* flies were previously shown to display a significantly higher level of ethanol self-administration (Das et al., 2013). Since increases in ethanol self-administration in *Drosophila* are likely a response to the hedonic properties of this drug (Devineni and Heberlein, 2009; Kaun et al., 2011; Xu et al., 2012), it is possible that the increased self-administration in the *Dunc13^P84200^*/+ heterozygotes is due to a naïve difference in the sensitivity to the neural effects of ethanol. To determine whether *Dunc13* activity is involved in ethanol intoxication, we examined heterozygotes for the *Dunc13^P84200^* mutation in a LOR assay (van der Linde et al., 2014). In the LOR assay, flies are exposed to ethanol vapor, which passively enters through their respiratory systems, increasing their internal alcohol concentrations (van der Linde et al., 2014). This leads to progressively more flies losing their righting-reflex with time. We use the T_1/2_, which is time needed for 50% of the flies to lose their righting reflex, to compare differences in the rate of intoxication (van der Linde et al., 2014).

**Figure 3.**
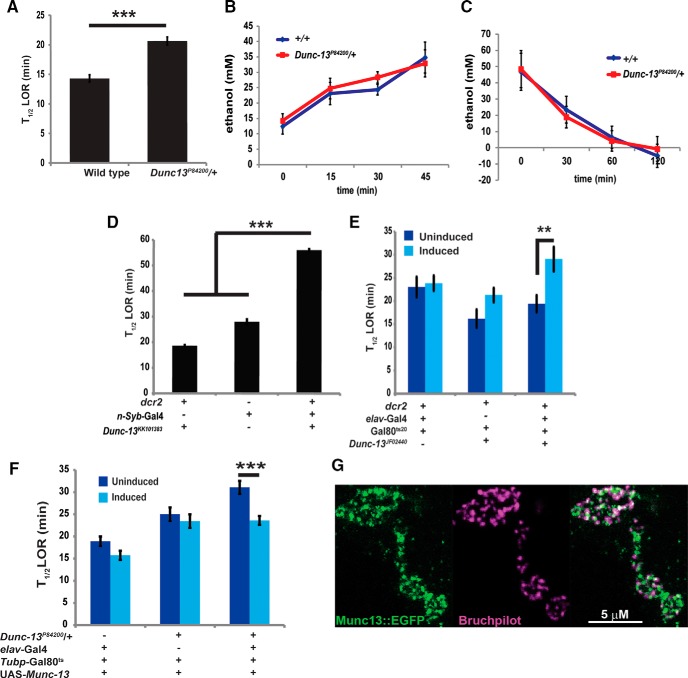
A reduction in Dunc13 activity leads to an increased resistance to ethanol sedation. ***A***, The *Dunc13^P84200^*/+ heterozygotes require a greater time to reach 50% LOR (T1/2 LOR) reflex levels (***p < 0.001, *n* = 17). ***B***, The concentration of ethanol was determined in *Dunc13^P84200^*/+ and control flies exposed to 50% ethanol vapor for 0, 15, 30, or 45 min; no significant differences were found (*p* > 0.05, *n* = 6). ***C***, The ability of *Dunc13^P84200^*/+ and control flies to metabolize ethanol was determined by first exposing flies to ethanol vapor for 45 min, and then by measuring the ethanol remaining in the flies 0, 30, 60, and 120 min after the exposure. No significant differences in ethanol metabolism were detected at each time point (*t* = 0.037, *p* > 0.05, *n* = 6). ***D***, The neural expression of the *Dunc13^KK101383^* RNAi transgene led to significantly slower T1/2 LOR compared to the genotype controls (***p < 0.05, *n* = 10). ***E***, The induced neural expression of the *Dunc13^JF02440^* RNAi transgenes also led to a significantly slower T1/2 LOR as compared to the within genotype control (*p* < 0.01, *n* = 8). Induction was accomplished with a 24 h, 30°C heat treatment, followed by a 3-h recovery period at room temperature. ***F***, Inducing the expression of a wild-type Munc13-1 cDNA for 48 h led to a significant decrease in LOR for the *Dunc13^P84200^*/+ flies (***p = 0.001, *N* = 9). Induction was accomplished with a 48 h, 30°C heat treatment, followed by a 3-h recovery period at room temperature. ***G***, Munc13-1::EGFP is colocalized with Bruchpilot, a protein localized to presynaptic active zones, in the presynaptic compartment of the larval neural muscular junction. All error bars are SEMs.

Genetically reducing *Dunc13* activity results in a reduced sensitivity to the sedative effects of ethanol. The *Dunc13^P84200^*/+ heterozygotes are significantly more resistant to the sedative effects of ethanol than wild-type controls (*t* = 7.246, *p* < 0.001; Fig. 3*A*). This increased resistance to ethanol vapor found in the *Dunc13^P84200^*/+ heterozygotes was not due to differences in the absorption of ethanol (*F*_(4,41)_ = 9.19, genotype difference *t* = 0.583, *p* = 0.563; Fig. 3*B*), or in the rate at which the heterozygotes metabolize the ethanol (*F*_(4,43)_ = 11.16, genotype difference *t* = 0.037, *p* = 0.971; Fig. 3*C*). Moreover, driving the expression of the *Dunc13^KK101383^* RNAi transgene throughout the nervous system using the *n-Syb*-Gal4 driver, also dramatically reduced the ethanol sedation sensitivity (*F*_(2,33)_ = 72.73, *p* < 0.0001; Fig. 3*D*). The T_1/2_ LOR for the experimental genotype, with Dunc13 RNAi being driven throughout the nervous system, was significantly longer than both the Gal4 driver control (*t* = 7.36, *p* < 0.0001) and the Dunc13 RNAi control genotypes (*t* = 19.18, *p* < 0.0001). The flies were viable, which suggested a partial knockdown of *Dunc13* in neural tissue. Lastly, we induced the expression of the *Dunc13^JF02440^* RNAi transgene postdevelopmentally in neurons with the *elav*-Gal4 and Gal80^ts20^ transgenes (McGuire et al., 2003). There were significant differences between groups in this experiment (*F*_(3,31)_ = 5.191, *p* < 0.005). A 24-h 30°C induction of *Dunc13* RNAi expression also leads to a significant decrease in ethanol sedation sensitivity compared to the within genotype control (*t* = 2.95, *p* = 0.011; Fig. 3*E*). Hence, reducing *Dunc13* activity through mutation or by using two nonoverlapping RNAi lines expressed in the nervous system by two independent neuronal drivers, all result in flies that are more resistant to the sedative effects of ethanol. The independence of these drivers and RNAi lines provide a strong indication that reducing Dunc13 activity, similar to the *Dunc13^P84200^*/+ haploinsufficent flies, produces a resistance to the sedative effects of ethanol. The ability of the induced *Dunc13^JF02440^* RNAi expression to decrease ethanol sedation sensitivity further suggests that this change results from a physiologic rather than a developmental response to reduced *Dunc13* activity, however, the RNAi transgene was expressed for 2 d in this experiment, which would permit homeostatic response to the reduced *Dunc13* activity.

We further verified the requirement for wild-type levels of *Dunc13* for normal sedation sensitivity by rescuing the haploinsufficiency of the *Dunc13^P84200^/+* heterozygotes. Previously, we had shown that a rat *Munc13-1::EGFP* cDNA was capable of rescuing the *Dunc13^P84200^*/*+* increased ethanol self-administration phenotype when expression was induced throughout the nervous system (Das et al., 2013). Herein, we examined whether *Munc13-1::EGFP* expression could also complement the *Dunc13^P84200^* haploinsufficiency in ethanol sedation (*F*_(3,56)_ = 9.875, *p* < 0.0001). The induced expression of this *Munc13-1::EGFP* transgene (48 h at 30°C) reduced the ethanol sedation sensitivity phenotype of *Dunc13^P84200^*/*+* females (*t* = 3.90, *p* = 0.001; Fig. 3*G*). The activity of Munc13-1 can therefore functionally complement the *Dunc13* activity required for normal ethanol sedation sensitivity. These data further indicate that ethanol sedation sensitivity is modulated by Dunc13/Munc13-1 activity.

We next examined whether *Munc13-1::EGFP* could also rescue the lethality of *Dunc13^P84200^* homozygotes. Two different UAS-*Munc13-1::EGFP* transgenes failed to rescue the late embryonic lethality of *Dunc13^P84200^* when driven by *Tubp*-Gal4, *elav*-Gal4, or *n-Syb*-Gal4, suggesting that *Munc13-1* may not be able to fully complement *Dunc13* neural functions (over 300 progeny of each genotype, with no viable homozygous *Dunc13^P84200^*adult progeny). One possible reason for the lack of full complementation could be due to problems in trafficking the relatively large Munc13-1::EFGP protein to the presynaptic compartment. To see whether the Munc13-1::GFP protein was localized to the presynaptic compartment in *Drosophila*, we examined the neuromuscular junction of 3^rd^ instar larvae with the genotype: UAS-*Munc13-1::GFP*/+; *nsyb*-Gal4/+. Munc13-1::EGFP was located within the presynaptic compartment in puncta. Bruchpilot, a protein localized to presynaptic active zones (Wagh et al., 2006), was found to be largely colocalized with Munc13-1::EGFP, although there exists some Munc13-1::EGFP puncta that are nonoverlapping with Bruchpilot signals and vice versa (Fig. 3*H*). Hence, the Munc13-1 was trafficked to presynaptic compartments.

## Discussion

The work that we present here identifies a new presynaptic mechanism for modulating ethanol sedation sensitivity. Ethanol binds to the C1 domain of Munc13-1 (Das et al., 2013), which inhibits DAG binding *in vitro*. This inhibition occurs within the physiologically intoxicating range of 25–50 mM ethanol (Majchrowicz, 1975). Inhibiting DAG binding will decrease the activity of Unc13 proteins by reducing the membrane localization of this protein, leading to an increased energy barrier for vesicle fusion and a reduction in synaptic vesicle release (Lackner et al., 1999; Basu et al., 2007). We found using *in vivo* optical physiologic approaches in *Drosophila* that intoxicating levels of ethanol did not significantly affect either membrane depolarization or Ca^2+^ influx within the presynaptic compartment of activated OR42b neurons, but synaptic vesicle fusion was dramatically inhibited in the neurons by these intoxicating levels of ethanol. Although our results do not show a significant effect on presynaptic Ca^2+^ levels after alcohol, we cannot rule out that the localization of Ca^2+^ microdomains was not impacted by ethanol. Nevertheless, our imaging results indicate that ethanol acts to inhibit synaptic vesicle release downstream of voltage-gated Ca^2+^ influx, which is consistent with ethanol inhibition of presynaptic activity through the reduction of DAG binding to Dunc13.

To verify a role for *Dunc13* in the physiologic and behavioral sensitivity to ethanol, we examined flies that had reduced levels of *Dunc13* activity. A simple prediction would be that these genetically sensitized flies, having already lost some *Dunc13* activity would be more sensitive to the effects of ethanol. However, the genetically sensitized flies were physiologically and behaviorally resistant to sedating concentrations of ethanol. The *Dunc13^P84200^*/*+* heterozygotes displayed reduced sensitivity to the acute effects of ethanol on synaptic vesicle fusion in the OR42b OSNs, and these flies also displayed reduced behavioral sensitivity to ethanol sedation. The reduced behavioral sensitivity to ethanol in the *Dunc13^P84200^*/*+* heterozygotes was partially reversed by neural expression of either *Dunc13* or *Munc13-1* cDNAs, indicating the phenotype was produced by a haploinsufficiency of *Dunc13*. Moreover, two independent and nonoverlapping *Dunc13* RNAi lines also displayed reduced sedation sensitivity. Together these data indicate that reducing *Dunc13* levels reduces the naïve responsiveness to the sedative effects of ethanol.

The functional complementation of *Munc13-1* for *Dunc13* haploinsufficiency in the LOR assay but not for the viability of the *Dunc13^P84200^* homozygotes indicates at least a partially conserved function between these orthologs in *Drosophila*. There can be several reasons for the inability of a *Munc13-1* cDNA to rescue the lethality of the homozygous *Dunc13^P84200^* flies, including differences in functional activity, expression levels, and protein localization. Munc13-1 contains several identified functional domains: C1 domain for DAG binding (Betz et al., 1998), CBS domain for binding calmodulin (Dimova et al., 2009), C2A domain for heterodimerization with RIM and regulating later steps in vesicle docking and priming (Betz et al., 2001; Camacho et al., 2017), C2B domain for binding phospholipid and VGCCs (Shin et al., 2010; Calloway et al., 2015), C2C domain, which may bind the plasma membrane (Rizo and Südhof, 1998), and MUN domain for binding syntaxin (Li et al., 2011). Although the C1, CBS, C2B, C2C and MUN domains of mammalian unc-13 and *Drosophila* unc-13 are highly conserved, Dunc13 lacks a conserved C2A domain (Aravamudan et al., 1999). It is currently unclear if the Dunc13 protein physically interacts with RIM in docked vesicles, but since this is a central interaction for vesicle priming it is likely this interaction is conserved in Drosophila. Interestingly, the C2A domain and the neighboring N-terminal sequences are necessary for the *C. elegans* unc-13 isoform L to be correctly located at the active zone (Hu et al., 2013; Zhou et al., 2013). We found that the Munc13-1::EGFP was localized to puncta in the larval neural muscular junction, although not completely overlapping with the active zone Bruchpilot protein.

### How does a reduction of *Dunc13* activity lead to a resistance to the sedative effects of ethanol?

The *Dunc13^P84200^*/+ haploinsufficiency and the neurally-expressed *Dunc13* RNAi lines likely mimic the initial effects of intoxicating concentrations of ethanol on this protein by genetically reducing its activity. The genetic or pharmacological reduction in Dunc13 activity would be expected to generate a widespread reduction in the size of the readily releasable pool (RRP; Augustin et al., 1999). This reduction in the RRP would produce synaptic depression, triggering a homeostatic response in the affected synapses (Davis and Goodman, 1998; Frank, 2014). Work from the *Drosophila* neural muscular junction has demonstrated two convergent mechanisms for homeostatic increases in presynaptic activity: potentiating Ca^2+^ influx through VGCCs and by increasing the RRP (Frank et al., 2009; Müller et al., 2012). In vertebrate central synapses, similar presynaptic homeostatic mechanisms have been discovered in which inhibition of activity leads to increases in both presynaptic Ca^2+^ influx and in synaptic vesicle release (Zhao et al., 2011). The presynaptic portion of this process may be largely driven by changes in cyclin-dependent kinase 5 (CDK5) activity (Kim and Ryan, 2010). Suppression of synaptic activity decreases CDK5 activity, which in turn promotes presynaptic Ca^2+^ influx and increases the resting vesicle pool available for promotion into the RRP (Kim and Ryan, 2010).

The homeostatic changes in synaptic efficacy brought about by ethanol reducing Dunc13 activity may mimic tolerance. Ethanol resistance and tolerance share a reduced response to this drug but differ in their ontogeny. Tolerance is a reduced responsiveness to alcohol brought about by previous exposure to this drug, whereas resistance refers to an innate difference in sensitivity to alcohol (Ghezzi and Atkinson, 2011; Rothenfluh et al., 2014). *Drosophila melanogaster* undergoes functional tolerance when exposed to high levels of alcohol, but does not develop metabolic tolerance (Scholz et al., 2000). This functional tolerance in *Drosophila* can be long lasting and dependent on epigenetic changes in gene expression and new protein synthesis (Berger et al., 2004; Cowmeadow et al., 2006; Engel et al., 2016).

There has been increasing evidence from *Drosophila* indicating that the development of functional tolerance is triggered by changes in presynaptic activity. Conditional alleles in the dynamin gene *shibire* (*shi*) can completely block the formation of rapid functional tolerance (Krishnan et al., 2012). At nonpermissive temperatures, the dominant negative *shi^ts^* alleles block synaptic vesicle recycling resulting in a depletion of the vesicle pool and a loss of presynaptic activity as well as other endocytosis-dependent events, such as receptor internalization (Kosaka and Ikeda, 1983). When this block in vesicle recycling was induced during sedation, there was a failure in the production of tolerance, while if it occurred after the initial binge exposure, there was no impact on tolerance, indicating a role for *shi* in the induction of tolerance (Krishnan et al., 2012). Interestingly, blocking neural activity during sedation using a *paralytic^ts^* conditional allele in a voltage-gated sodium channel or temperature-sensitive alleles of the *comatose* NSF protein did not block the formation of functional tolerance. These data suggest that dynamics in the presynaptic vesicle pool during ethanol sedation, and not changes in presynaptic activity per se, are required for tolerance formation (Krishnan et al., 2012).

Alcohol exposures that induce functional tolerance also bring about long-lasting changes in the levels of several presynaptic proteins that are required for the induction of functional tolerance (Ghezzi et al., 2013). Some of these transcriptional changes necessary for chronic tolerance require the activity of the Sir2 histone deacetylase (Engel et al., 2016). These Sir2-dependent epigenetic changes regulate the activity of presynaptic proteins including Synapsin and also likely the *cacophony* voltage-gated Ca^2+^ channel and the *cdk5* kinase, which are known to have important roles in presynaptic homeostatic responses to reduced synaptic activity (Davis, 2013; Frank, 2014). Mutants in Synapsin are defective in tolerance formation, but not in naïve sedation (Godenschwege et al., 2004; Engel et al., 2016). Moreover, increases in the level of the slowpoke BK channel are also required for the development of functional tolerance (Cowmeadow et al., 2005; Cowmeadow et al., 2006; Li et al., 2013). Increases in *slowpoke* increase the ability for hire frequency firing (Ghezzi et al., 2010). This increase in high-frequency firing may reverse synaptic depression by facilitating the ability of Ca^+2^ to increase the rate at which the RRP is replenished (Wang and Kaczmarek, 1998).

In sum, there is strong evidence for specific homeostatic changes in presynaptic activity underlying ethanol tolerance, and an initial trigger for these changes may be ethanol inhibiting the binding of DAG to the C1 domain of Dunc13. A testable prediction from this hypothesis for the inhibition of Dunc13 as an initial event in the formation of tolerance is that compounds or mutations, such as the Munc13-1 E_582_A, that inhibit ethanol binding to the C1 domain will reduce tolerance formation (Das et al., 2013). However, for these reagents to be useful in elucidating the role of Dunc13 inhibition in tolerance formation, they need to have a limited effect on Dunc13/Munc13-1 activity in the absence of ethanol. Otherwise they may lead to the same homeostatic changes in naive animals found after exposure to intoxicating levels of ethanol.

## Synthesis

Reviewing Editor: Karen Szumlinski, University of California at Santa Barbara Decisions are customarily a result of the Reviewing Editor and the peer reviewers coming together and discussing their recommendations until a consensus is reached. When revisions are invited, a fact-based synthesis statement explaining their decision and outlining what is needed to prepare a revision will be listed below. The following reviewer(s) agreed to reveal their identity: Adrian Rothenfluh, Jill Bettinger

Upon consultation with 2 expert reviewers, I have reached the decision of “Revise-Reviewing Editor Review Only”. Both reviewers found the report to be very well written, but have a few suggestions for improvement. While both reviewers thought that the inclusion of data from the DUNC13-E582A transgenic fly would render the conclusions stronger, we are all in agreement that including such experiments are not necessary for the report, but some discussion related to the utility of such a mutant line is warranted. Please find below the specific comments of each reviewer. I look forward to reviewing your revised report.

Reviewer 1:

The MS eN-NWR-0125-18 looks at the contribution of Dunc13 to ethanol-induced responses. This includes looking at the effect of ethanol on neuron physiology by various imaging means, and comparing the response in Dunc13 hemizygotes. Authors also show effects of hemizygotes on behavior, as well as conditional knock-down and rescue. Lastly, their data suggest an overlap of the EtOH and DAG binding site in Munc. This is a nice, well-written paper, and I only have few suggestions for improvement.

Minor comments:

The interpretation of Munc13's role in tolerance and homeostatic upregulation are not supported by any data, and are better suited for a more speculative venue, like a review (or the end of the discussion, as done in the MS, but not the abstract).

L.247: “flies were LESS SENSITIVE to this presynaptic inhibition” would hew closer to the data than saying ‘resistant’.

l.298: sentence misses a subject

Other interpretations of the non-rescue with Munc13-1 are: 1) necessary maternal contribution, 2) requirement for exact levels, i.e. too much is lethal as well. Both testable, if somewhat low yield.

l.368 is belied by Figure 2G, where EtOH has a strong effect, but Dunc13/+ alone (no EtOH) does not.

I think the presynaptic interpretation of the shibire results are a bit overinterpreted, after all dynamin is not only involved in SV endocytosis, but neurotransmitter receptor endocytosis too. In fact, the postsynaptic protein Homer is involved in Drosophila alcohol tolerance (Urizar 2007), arguing against the emphasis of the pre-synapse.

I couldn't find the methods for the temperature regimen used in Fig.3E,F. It should be included in the Figure, or Legend. Temperature in and of itself has an effect on flies sensitivity to ethanol-induced sedation (e.g. Eddison 2011, and others too), and it's a bit surprising to see little evidence of that in these Figures. Since, according to the methods anyway, all flies were outcrossed to Canton-S (even ci[D], and the JF-RNAi marked with y+ v+ - is that true?), the right statistical comparisons would be between the genotypes within temperature.

Reviewer 2:

It is important to note that the definitive experiment is not done here: If the E582 mutant doesn't have the ethanol-induced inhibition of Dunc13, then the authors could directly test if the ethanol-induced inhibition of presynaptic vesicle release is dependent on Dunc13 in transgenic animals in which this mutant is either knocked in or transgenically expressed in the mutant. That's a reasonably involved experiment, but it would substantially strengthen the conclusions.

The Methods section describing the in vitro work is really much too limited. I had to look up the Das 2013 paper to understand the details. The Methods section for this experiment should be much more comprehensive.

A few wording concerns:

Saying that ethanol ‘directly’ inhibits presynaptic vesicle release is unsupported. A finding of direct inhibition in vivo would require you to show conclusively that the effect on Dunc13 is the cause of the change in presynaptic vesicle release. As it is, it's a good bet, but not shown. EtOH may inhibit release independent of a role for Dunc13.

I object to the ‘hedonic’ properties of the drug in discussions of Drosophila. It's anthropomorphic and not necessary. Furthermore, it's conjecture. You can use pharmacodynamic.

## References

[B1] Akerboom J, Chen T-W, Wardill TJ, Tian L, Marvin JS, Mutlu S, Calderón NC, Esposti F, Borghuis BG, Sun XR (2012) Optimization of a GCaMP calcium indicator for neural activity imaging. J Neurosci 32:13819–13840. 10.1523/JNEUROSCI.2601-12.2012 23035093 PMC3482105

[B2] Aravamudan B, Fergestad T, Davis WS, Rodesch CK, Broadie K (1999) *Drosophila* UNC-13 is essential for synaptic transmission. Nat Neurosci 2:965–971. 10.1038/14764 10526334

[B3] Augustin I, Rosenmund C, Südhof TC, Brose N (1999) Munc13-1 is essential for fusion competence of glutamatergic synaptic vesicles. Nature 400:457 10.1038/22768 10440375

[B4] Basu J, Betz A, Brose N, Rosenmund C (2007) Munc13-1 C1 domain activation lowers the energy barrier for synaptic vesicle fusion. J Neurosci 27:1200–1210. 10.1523/JNEUROSCI.4908-06.2007 17267576 PMC6673179

[B5] Berger KH, Heberlein U, Moore MS (2004) Rapid and chronic: two distinct forms of ethanol tolerance in *Drosophila*. Alcohol Clin Exp Res 28:1469–1480. 15597078 10.1097/01.alc.0000141817.15993.98

[B6] Betz A, Okamoto M, Benseler F, Brose N (1997) Direct interaction of the rat unc-13 homologue Munc13-1 with the N terminus of syntaxin. J Biol Chem 272:2520–2526. 10.1074/jbc.272.4.2520 8999968

[B7] Betz A, Ashery U, Rickmann M, Augustin I, Neher E, Südhof TC, Rettig J, Brose N (1998) Munc13-1 is a presynaptic phorbol ester receptor that enhances neurotransmitter release. Neuron 21:123–136. 10.1016/S0896-6273(00)80520-6 9697857

[B8] Betz A, Thakur P, Junge HJ, Ashery U, Rhee JS, Scheuss V, Rosenmund C, Rettig J, Brose N (2001) Functional interaction of the active zone proteins Munc13-1 and RIM1 in synaptic vesicle priming. Neuron 30:183–196. 10.1016/S0896-6273(01)00272-0 11343654

[B9] Bischof J, Maeda RK, Hediger M, Karch F, Basler K (2007) An optimized transgenesis system for *Drosophila* using germ-line-specific phiC31 integrases. Proc Natl Acad Sci USA 104:3312–3317. 10.1073/pnas.0611511104 17360644 PMC1805588

[B10] Bukiya AN, Kuntamallappanavar G, Edwards J, Singh AK, Shivakumar B, Dopico AM (2014) An alcohol-sensing site in the calcium- and voltage-gated, large conductance potassium (BK) channel. Proc Natl Acad Sci USA 111:9313–9318. 10.1073/pnas.1317363111 24927535 PMC4078796

[B11] Calloway N, Gouzer G, Xue M, Ryan TA (2015) The active-zone protein Munc13 controls the use-dependence of presynaptic voltage-gated calcium channels. Elife 4. 10.7554/eLife.07728 PMC4525472 26196145

[B12] Camacho M, Basu J, Trimbuch T, Chang S, Pulido-Lozano C, Chang SS, Duluvova I, Abo-Rady M, Rizo J, Rosenmund C (2017) Heterodimerization of Munc13 C2A domain with RIM regulates synaptic vesicle docking and priming. Nat Commun 8:15293. 10.1038/ncomms15293 28489077 PMC5436228

[B13] Cao G, Platisa J, Pieribone VA, Raccuglia D, Kunst M, Nitabach MN (2013) Genetically targeted optical electrophysiology in intact neural circuits. Cell 154:904–913. 10.1016/j.cell.2013.07.027 23932121 PMC3874294

[B14] Couto A, Alenius M, Dickson BJ (2005) Molecular, anatomical, and functional organization of the *Drosophila* olfactory system. Curr Biol 15:1535–1547. 10.1016/j.cub.2005.07.034 16139208

[B15] Cowmeadow RB, Krishnan HR, Atkinson NS (2005) The slowpoke gene is necessary for rapid ethanol tolerance in *Drosophila*. Alcohol Clin Exp Res 29:1777–1786. 16269907 10.1097/01.alc.0000183232.56788.62

[B16] Cowmeadow RB, Krishnan HR, Ghezzi A, Al'Hasan YM, Wang YZ, Atkinson NS (2006) Ethanol tolerance caused by slowpoke induction in *Drosophila*. Alcohol Clin Exp Res 30:745–753. 10.1111/j.1530-0277.2006.00087.x 16634842

[B17] Das J, Xu S, Pany S, Guillory A, Shah V, Roman GW (2013) The pre-synaptic Munc13-1 binds alcohol and modulates alcohol self-administration in *Drosophila*. J Neurochem 126:715–726. 10.1111/jnc.12315 23692447 PMC3766433

[B18] Davies AG, Pierce-Shimomura JT, Kim H, VanHoven MK, Thiele TR, Bonci A, Bargmann CI, McIntire SL (2003) A central role of the BK potassium channel in behavioral responses to ethanol in *C. elegans*. Cell 115:655–666. 14675531 10.1016/s0092-8674(03)00979-6

[B19] Davis GW (2013) Homeostatic signaling and the stabilization of neural function. Neuron 80:718–728. 10.1016/j.neuron.2013.09.044 24183022 PMC3856728

[B20] Davis GW, Goodman CS (1998) Synapse-specific control of synaptic efficacy at the terminals of a single neuron. Nature 392:82–86. 10.1038/32176 9510251

[B21] Davis SJ, Scott LL, Hu K, Pierce-Shimomura JT (2014) Conserved single residue in the BK potassium channel required for activation by alcohol and intoxication in *C. elegans* . J Neurosci 34:9562–9573. 10.1523/JNEUROSCI.0838-14.2014 25031399 PMC4099540

[B22] Devineni AV, Heberlein U (2009) Preferential ethanol consumption in *Drosophila* models features of addiction. Curr Biol 19:2126–2132. 10.1016/j.cub.2009.10.070 20005106 PMC2805771

[B23] Diamond I, Gordon AS (1997) Cellular and molecular neuroscience of alcoholism. Physiol Rev 77:1–20. 10.1152/physrev.1997.77.1.1 9016298

[B24] Dick DM, Jones K, Saccone N, Hinrichs A, Wang JC, Goate A, Bierut L, Almasy L, Schuckit M, Hesselbrock V (2006) Endophenotypes successfully lead to gene identification: results from the collaborative study on the genetics of alcoholism. Behav Genet 36:112–126. 10.1007/s10519-005-9001-3 16341909

[B25] Dimova K, Kalkhof S, Pottratz I, Ihling C, Rodriguez-Castaneda F, Liepold T, Griesinger C, Brose N, Sinz A, Jahn O (2009) Structural insights into the calmodulin-Munc13 interaction obtained by cross-linking and mass spectrometry. Biochemistry 48:5908–5921. 10.1021/bi900300r 19492809

[B26] Engel GL, Marella S, Kaun KR, Wu J, Adhikari P, Kong EC, Wolf FW (2016) Sir2/Sirt1 links acute inebriation to presynaptic changes and the development of alcohol tolerance, preference, and reward. J Neurosci 36:5241–5251. 10.1523/JNEUROSCI.0499-16.2016 27170122 PMC4863060

[B27] Fehr C, Shirley RL, Crabbe JC, Belknap JK, Buck KJ, Phillips TJ (2005) The syntaxin binding protein 1 gene (Stxbp1) is a candidate for an ethanol preference drinking locus on mouse chromosome 2. Alcohol Clin Exp Res 29:708–720. 15897714 10.1097/01.alc.0000164366.18376.ef

[B28] Frank CA (2014) Homeostatic plasticity at the *Drosophila* neuromuscular junction. Neuropharmacology 78:63–74. 10.1016/j.neuropharm.2013.06.015 23806804 PMC3830618

[B29] Frank CA, Pielage J, Davis GW (2009) A presynaptic homeostatic signaling system composed of the Eph receptor, ephexin, Cdc42, and Ca V 2.1 calcium channels. Neuron 61:556–569. 10.1016/j.neuron.2008.12.028 19249276 PMC2699049

[B30] Gao Q, Yuan B, Chess A (2000) Convergent projections of *Drosophila* olfactory neurons to specific glomeruli in the antennal lobe. Nat Neurosci 3:780–785. 10.1038/77680 10903570

[B31] Ghezzi A, Atkinson NS (2011) Homeostatic control of neural activity: a *Drosophila* model for drug tolerance and dependence. Int Rev Neurobiol 99:23–50. 10.1016/B978-0-12-387003-2.00002-1 21906535 PMC4862361

[B32] Ghezzi A, Pohl JB, Wang Y, Atkinson NS (2010) BK channels play a counter-adaptive role in drug tolerance and dependence. Proc Natl Acad Sci USA 107:16360–16365. 10.1073/pnas.1005439107 20798347 PMC2941274

[B33] Ghezzi A, Krishnan HR, Lew L, Prado FJ 3rd, Ong DS, Atkinson NS (2013) Alcohol-induced histone acetylation reveals a gene network involved in alcohol tolerance. PLoS Genet 9:e1003986 10.1371/journal.pgen.1003986 24348266 PMC3861128

[B77] Godenschwege TA, Reisch D, Diegelmann S, Eberle K, Funk N, Heisenberg M, ... & Nikitina EA (2004) Flies lacking all synapsins are unexpectedly healthy but are impaired in complex behaviour. European Journal of Neuroscience 20(3):611–622. 15255973 10.1111/j.1460-9568.2004.03527.x

[B34] Graham ME, Edwards MR, Holden-Dye L, Morgan A, Burgoyne RD, Barclay JW (2009) UNC-18 modulates ethanol sensitivity in *Caenorhabditis elegans* . Mol Biol Cell 20:43–55. 10.1091/mbc.E08-07-0689 18923141 PMC2613081

[B35] Hallem EA, Carlson JR (2006) Coding of odors by a receptor repertoire. Cell 125:143–160. 10.1016/j.cell.2006.01.050 16615896

[B36] Harris RA, Trudell JR, Mihic SJ (2008) Ethanol’s molecular targets. Sci Signal 1:re7. 10.1126/scisignal.128re7 18632551 PMC2671803

[B37] Hu Z, Tong XJ, Kaplan JM (2013) UNC-13L, UNC-13S, and Tomosyn form a protein code for fast and slow neurotransmitter release in *Caenorhabditis elegans* . Elife 2:e00967 10.7554/eLife.00967 23951547 PMC3743133

[B38] Kapfhamer D, Bettinger JC, Davies AG, Eastman CL, Smail EA, Heberlein U, McIntire SL (2008) Loss of RAB-3/A in *Caenorhabditis elegans* and the mouse affects behavioral response to ethanol. Genes Brain Behav 7:669–676. 10.1111/j.1601-183X.2008.00404.x 18397381 PMC3526002

[B39] Kaun KR, Azanchi R, Maung Z, Hirsh J, Heberlein U (2011) A *Drosophila* model for alcohol reward. Nat Neurosci 14:612–619. 10.1038/nn.2805 21499254 PMC4249630

[B40] Kim SH, Ryan TA (2010) CDK5 serves as a major control point in neurotransmitter release. Neuron 67:797–809. 10.1016/j.neuron.2010.08.003 20826311 PMC2939042

[B41] Koob GF, Bloom FE (1988) Cellular and molecular mechanisms of drug dependence. Science 242:715–723. 2903550 10.1126/science.2903550

[B42] Kosaka T, Ikeda K (1983) Possible temperature‐dependent blockage of synaptic vesicle recycling induced by a single gene mutation in *Drosophila* . Dev Neurobiol 14:207–225. 10.1002/neu.480140305 6304244

[B43] Krishnan HR, Al-Hasan YM, Pohl JB, Ghezzi A, Atkinson NS (2012) A role for dynamin in triggering ethanol tolerance. Alcohol Clin Exp Res 36:24–34. 10.1111/j.1530-0277.2011.01587.x 21797886 PMC3208067

[B44] Lackner MR, Nurrish SJ, Kaplan JM (1999) Facilitation of synaptic transmission by EGL-30 G q α and EGL-8 PLCβ: DAG binding to UNC-13 is required to stimulate acetylcholine release. Neuron 24:335–346. 10.1016/S0896-6273(00)80848-X 10571228

[B45] Li W, Ma C, Guan R, Xu Y, Tomchick DR, Rizo J (2011) The crystal structure of a Munc13 C-terminal module exhibits a remarkable similarity to vesicle tethering factors. Structure 19:1443–1455. 10.1016/j.str.2011.07.012 22000513 PMC3197213

[B46] Li X, Ghezzi A, Pohl JB, Bohm AY, Atkinson NS (2013) A DNA element regulates drug tolerance and withdrawal in *Drosophila*. PLoS One 8:e75549. 10.1371/journal.pone.0075549 24086565 PMC3781064

[B47] Liu Y, Hunt WA, eds (1999) The drunken synapse: studies of alcohol related disorders. New York: Klewer Academic/Plenum Publishing Corp.

[B48] Wang LY, Kaczmarek LK (1998) High-frequency firing helps replenish the readily releasable pool of synaptic vesicles. Nature 394:384. 10.1038/28645 9690475

[B49] Majchrowicz E (1975) Induction of physical dependence upon ethanol and the associated behavioral changes in rats. Psychopharmacologia 43:245–254. 1237914 10.1007/BF00429258

[B50] Maldve RE, Chen X, Zhang TA, Morrisett RA (2004) Ethanol selectively inhibits enhanced vesicular release at excitatory synapses: real-time visualization in intact hippocampal slices. Alcohol Clin Exp Res 28:143–152. 10.1097/01.ALC.0000106304.39174.AD 14745313

[B51] Mayfield R, Harris R, Schuckit M (2008) Genetic factors influencing alcohol dependence. Br J Pharmacol 154:275–287. 10.1038/bjp.2008.88 18362899 PMC2442454

[B52] McGuire SE, Le PT, Osborn AJ, Matsumoto K, Davis RL (2003) Spatiotemporal rescue of memory dysfunction in *Drosophila* . Science 302:1765–1768. 10.1126/science.1089035 14657498

[B53] Müller M, Liu KSY, Sigrist SJ, Davis GW (2012) RIM controls homeostatic plasticity through modulation of the readily-releasable vesicle pool. J Neurosci 32:16574–16585. 10.1523/JNEUROSCI.0981-12.2012 23175813 PMC3523185

[B54] Ojelade SA, Acevedo SF, Kalahasti G, Rodan AR, Rothenfluh A (2015) RhoGAP18B isoforms act on distinct Rho-family GTPases and regulate behavioral responses to alcohol via cofilin. PLoS One 10:e0137465. 10.1371/journal.pone.0137465 26366560 PMC4569326

[B55] Pany S, Das J (2015) Alcohol binding in the C1 (C1A+C1B) domain of protein kinase C epsilon. Biochim Biophys Acta 1850:2368–2376. 10.1016/j.bbagen.2015.07.005 26210390 PMC4586410

[B56] Reiff DF, Ihring A, Guerrero G, Isacoff EY, Joesch M, Nakai J, Borst A (2005) In vivo performance of genetically encoded indicators of neural activity in flies. J Neurosci 25:4766–4778. 15888652 10.1523/JNEUROSCI.4900-04.2005 PMC1464576

[B57] Rhee JS, Betz A, Pyott S, Reim K, Varoqueaux F, Augustin I, Hesse D, Südhof TC, Takahashi M, Rosenmund C, Brose N (2002) Beta phorbol ester- and diacylglycerol-induced augmentation of transmitter release is mediated by Munc13s and not by PKCs. Cell 108:121–133. 10.1016/S0092-8674(01)00635-3 11792326

[B58] Rizo J, Südhof TC (1998) C2-domains, structure and function of a universal Ca2+-binding domain. J Biol Chem 273:15879–15882. 9632630 10.1074/jbc.273.26.15879

[B59] Rizo J, Xu J (2015) The synaptic vesicle release machinery. Annu Rev Biophys 44:339–367. 10.1146/annurev-biophys-060414-034057 26098518

[B60] Roman G, He J, Davis RL (1999) New series of *Drosophila* expression vectors suitable for behavioral rescue. Biotechniques 27:54–56. 10407662 10.2144/99271bm09

[B61] Rothenfluh A, Troutwine B, Ghezzi A, Atkinson N (2014) The genetics of alcohol responses of invertebrate model systems In: Neurobiology of alcohol dependence, pp 467-495. New York: Elsevier.

[B62] Sailer CA, Kaufmann WA, Kogler M, Chen L, Sausbier U, Ottersen OP, Ruth P, Shipston MJ, Knaus HG (2006) Immunolocalization of BK channels in hippocampal pyramidal neurons. Eur J Neurosci 24:442–454. 10.1111/j.1460-9568.2006.04936.x 16903852

[B63] Salvatore JE, Gottesman II, Dick DM (2015) Endophenotypes for alcohol use disorder: an update on the field. Curr Addict Rep 2:76–90. 10.1007/s40429-015-0046-y 26236574 PMC4520241

[B64] Scholz H, Ramond J, Singh CM, Heberlein U (2000) Functional ethanol tolerance in *Drosophila* . Neuron 28:261–271. 11086999 10.1016/s0896-6273(00)00101-x

[B65] Schuckit MA (1980) Biological markers: metabolism and acute reactions to alcohol in sons of alcoholics. Pharmacol Biochem Behav 13:9–16. 10.1016/S0091-3057(80)80003-7 7243837

[B66] Schuckit MA (1994) Alcohol sensitivity and dependence. EXS 71:341–348. 8032165 10.1007/978-3-0348-7330-7_34

[B67] Schuckit MA, Tsuang JW, Anthenelli RM, Tipp JE, Nurnberger JI Jr (1996) Alcohol challenges in young men from alcoholic pedigrees and control families: a report from the COGA project. J Stud Alcohol 57:368–377. 8776678 10.15288/jsa.1996.57.368

[B68] Shin OH, Lu J, Rhee JS, Tomchick DR, Pang ZP, Wojcik SM, Camacho-Perez M, Brose N, Machius M, Rizo J, Rosenmund C, Südhof TC (2010) Munc13 C2B domain is an activity-dependent Ca2+ regulator of synaptic exocytosis. Nat Struct Mol Biol 17:280–288. 10.1038/nsmb.1758 20154707 PMC2916016

[B69] Urizar NL, Yang Z, Edenberg HJ, Davis RL (2007) *Drosophila* homer is required in a small set of neurons including the ellipsoid body for normal ethanol sensitivity and tolerance. J Neurosci 27:4541–4551. 10.1523/JNEUROSCI.0305-07.2007 17460067 PMC6672997

[B70] van der Linde K, Fumagalli E, Roman G, Lyons LC (2014) The FlyBar: administering alcohol to flies. J Vis Exp [Please provide volume number and page range for van der Linde et al (2014).] 10.3791/50442 PMC4193141 24895004

[B71] Wagh DA, Rasse TM, Asan E, Hofbauer A, Schwenkert I, Dürrbeck H, Buchner S, Dabauvalle MC, Schmidt M, Qin G, Wichmann C, Kittel R, Sigrist SJ, Buchner E (2006) Bruchpilot, a protein with homology to ELKS/CAST, is required for structural integrity and function of synaptic active zones in *Drosophila* . Neuron 49:833–844. 10.1016/j.neuron.2006.02.008 16543132

[B72] Xu S, Chan T, Shah V, Zhang S, Pletcher SD, Roman G (2012) The propensity for consuming ethanol in *Drosophila* requires rutabaga adenylyl cyclase expression within mushroom body neurons. Genes Brain Behav 11:727–739. 10.1111/j.1601-183X.2012.00810.x 22624869 PMC3404234

[B73] Yuste R, Miller RB, Holthoff K, Zhang S, Miesenböck G (2000) Synapto-pHluorins: chimeras between pH-sensitive mutants of green fluorescent protein and synaptic vesicle membrane proteins as reporters of neurotransmitter release. Methods Enzymol 327:522–546. 11045007 10.1016/s0076-6879(00)27300-x

[B74] Zhang S, Roman G (2013) Presynaptic inhibition of gamma lobe neurons is required for olfactory learning in *Drosophila* . Curr Biol 23:2519–2527. 10.1016/j.cub.2013.10.043 24291093

[B75] Zhao C, Dreosti E, Lagnado L (2011) Homeostatic synaptic plasticity through changes in presynaptic calcium influx. J Neurosci 31:7492–7496. 10.1523/JNEUROSCI.6636-10.2011 21593333 PMC3124754

[B76] Zhou K, Stawicki TM, Goncharov A, Jin Y (2013) Position of UNC-13 in the active zone regulates synaptic vesicle release probability and release kinetics. Elife 2:e01180. 10.7554/eLife.01180 24220508 PMC3821175

